# *NOTCH2 *in breast cancer: association of SNP rs11249433 with gene expression in ER-positive breast tumors without *TP53 *mutations

**DOI:** 10.1186/1476-4598-9-113

**Published:** 2010-05-19

**Authors:** Yi-Ping Fu, Hege Edvardsen, Alpana Kaushiva, Juan P Arhancet, Tiffany M Howe, Indu Kohaar, Patricia Porter-Gill, Anushi Shah, Hege Landmark-Høyvik, Sophie D Fosså, Stefan Ambs, Bjørn Naume, Anne-Lise Børresen-Dale, Vessela N Kristensen, Ludmila Prokunina-Olsson

**Affiliations:** 1 Laboratory of Translational Genomics, Division of Cancer Epidemiology and Genetics, National Cancer Institute, National Institutes of Health, Bethesda, MD, USA; 2 Department of Genetics, Institute for Cancer Research, Oslo University Hospital Radiumhospitalet, Montebello, Oslo, Norway; 3 Faculty Division of Medicine, Radiumhospitalet, The Norwegian Radium Hospital, University of Oslo, Oslo, Norway; 4 Laboratory of Human Carcinogenesis, Center for Cancer Research, National Cancer Institute, National Institutes of Health, Bethesda, MD, USA; 5 Department of Clinical Cancer Research, Cancer Clinic, Oslo University Hospital Radiumhospitalet, Oslo, Norway; 6 Department of Oncology, Cancer Clinic, Oslo University Hospital Radiumhospitalet, Oslo, Norway; 7 Institute for Clinical Epidemiology and Molecular Biology (EpiGen), Faculty of Medicine, University of Oslo, Oslo, Norway

## Abstract

**Background:**

A recent genome-wide association study (GWAS) has identified a single nucleotide polymorphism (SNP) rs11249433 in the 1p11.2 region as a novel genetic risk factor for breast cancer, and this association was stronger in patients with estrogen receptor (ER)^+ ^versus ER^- ^cancer.

**Results:**

We found association between SNP rs11249433 and expression of the *NOTCH2 *gene located in the 1p11.2 region. Examined in 180 breast tumors, the expression of *NOTCH2 *was found to be lowest in tumors with *TP53 *mutations and highest in *TP53 *wild-type/ER^+ ^tumors (p = 0.0059). In the latter group, the *NOTCH2 *expression was particularly increased in carriers of the risk genotypes (AG/GG) of rs11249433 when compared to the non-risk AA genotype (p = 0.0062). Similar association between *NOTCH2 *expression and rs11249433 was observed in 60 samples of purified monocytes from healthy controls (p = 0.015), but not in total blood samples from 302 breast cancer patients and 76 normal breast tissue samples. We also identified the first possible dominant-negative form of *NOTCH2*, a truncated version of *NOTCH2 *consisting of only the extracellular domain.

**Conclusion:**

This is the first study to show that the expression of *NOTCH2 *differs in subgroups of breast tumors and by genotypes of the breast cancer-associated SNP rs11249433. The NOTCH pathway has key functions in stem cell differentiation of ER^+ ^luminal cells in the breast. Therefore, increased expression of *NOTCH2 *in carriers of rs11249433 may promote development of ER^+ ^luminal tumors. Further studies are needed to investigate possible mechanisms of regulation of *NOTCH2 *expression by rs11249433 and the role of *NOTCH2 *splicing forms in breast cancer development.

## Background

Several genome-wide association studies (GWAS) in individuals of European ancestry have identified novel genomic regions associated with risk of breast cancer (BC) [1-7]. One of the associated variants was a single nucleotide polymorphism (SNP) rs11249433 located within the pericentromeric region on chromosome 1p11.2 [4]. The association was found to be stronger with estrogen receptor (ER)^+ ^than with ER^- ^cancer [4]. Decreased genetic recombination rates within pericentromeric regions can contribute to large linkage disequilibrium (LD) blocks [8]. As a result, rs11249433 might be in LD with many other variations located over a large distance. The region surrounding rs11249433 is poorly covered in the International HapMap project [9] due to a high degree of segmental duplications that map to both sides of centromere on chromosome 1. However, rs11249433 itself is located in a region uniquely mapped to 1p11.2. Given the complicated genomic landscape, we did not perform fine-mapping or resequencing studies of this region, but sought to identify a possible molecular phenotype functionally important for breast carcinogenesis and related to this genetic association.

The heterogeneity of BC includes diverse molecular characteristics, and variations in clinical outcomes and treatment responses. BC patients can be categorized into different groups based on cellular morphology and expression of estrogen receptor (ER), progesterone receptor (PR), and human epidermal growth factor receptor 2 (HER-2) [10]. Mutational status of *TP53*, a tumor suppressor gene, is also of strong prognostic significance for BC [11]. Previous findings from mRNA expression profiling have identified several molecular categories of BC, such as luminal-A, luminal-B, normal breast-like, HER-2 positive and basal-like types [12-14]. These categories represent clinically distinct patient groups [13,14], and correspond to different breast tumor subtypes with respect to ER and *TP53 *mutation status: ER^+ ^tumors are generally identified as luminal-like subtype [14], while tumors with *TP53 *mutations usually belong to the basal-like type [15].

In this study, we observed a distinct expression profile of the *NOTCH2 *gene in various tumor subtypes. Furthermore, the association between rs11249433 and increased expression of *NOTCH2 *was found in patients with ER^+ ^breast tumors without *TP53 *mutations. We also identified a unique splicing form of *NOTCH2 *consisting of only the extracellular domain and thus predicted to have a dominant-negative function compared to the full-length form of the gene.

## Results and Discussion

### *NOTCH2 *as a candidate gene for BC risk in the 1p11.2 region

**Figure 1:**
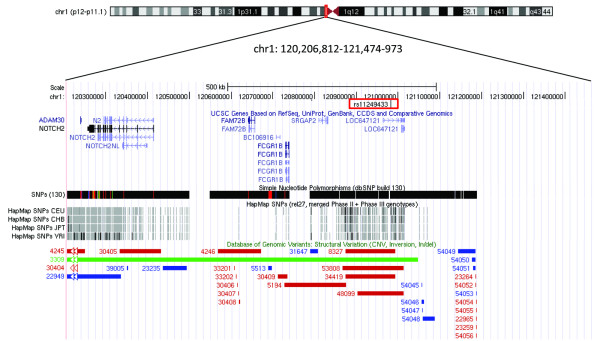
**Genomic organization of the 1p11.2 region surrounding the BC-associated rs11249433**. The map is based on genome build hg18 http://genome.ucsc.edu/ and shows genes, dbSNPs(130), HapMap SNPs (rel27) and structural variations.

Several studies have shown that cancer-associated SNPs identified by GWAS are regulatory variants that can be linked to altered gene expression [16-19]. Working under the hypothesis that the BC-associated rs11249433 may regulate mRNA expression of genes in the surrounding region, we analyzed microarray data for the expression of five genes located within a 1 Mb region telomeric from rs11249433 (*ADAM30*, *NOTCH2, NOTCH2NL, FAM72B*, and *SRGAP2*). There were no genes between this SNP and the centromere (Figure 1). Two other genes from this region, *LOC647121*, a non-coding pseudogene, and *FCGR1B*, which belongs to a family of highly similar genes, were not present on the array and thus were not examined here. Based on expression analysis in 108 breast tumor samples, there was a suggestive association between rs11249433 risk genotypes (AG and GG) and increased expression of the Notch homolog 2 gene (*NOTCH2*, p = 0.054) and decreased expression of the Notch homolog 2 N-terminal-like gene (*NOTCH2NL*, p = 0.038) (Table 1). The four known NOTCH proteins (NOTCH1 - 4) are highly conserved transmembrane receptors found in all multicellular organisms [20]. Interactions between membrane-bound ligands Jagged1, Jagged2, Delta1 and the extracellular domains of NOTCH proteins trigger a cascade of proteolytic cleavages that result in a release of the notch intracellular domain (NICD) and induced expression of target genes involved in response to hypoxia, stem cell maturation, migration and inhibition of differentiation [20-22]. The NOTCH signaling pathway has been well-studied as one of the underlying mechanisms and potential therapeutic targets for BC [23,24]. However, the majority of information about the NOTCH pathway is related to *NOTCH1* gene, while the potential role of *NOTCH2 *gene is still not well established. Interestingly, a recent report indicated that a higher expression level of *NOTCH2 *can be found in low-grade/well-differentiated breast tumors as compared to poorly-differentiated tumors [25].

**Table 1:** Results of microarray analysis for 5 genes within 1 Mb from rs11249433 in the 1p11.2 region.

Gene/SNP	Position, bp^+^	rs11249433	N	Fold*	95%CI	p-value*
*ADAM30*	chr1: 120,237,679 - 120,240,636	AA	35	1.00	0.90 - 1.11	0.378
		AG	41	1.06	0.96 - 1.16	
		GG	13	0.93	0.80 - 1.09	
						
*NOTCH2*	chr1: 120,279,260 - 120,413,799	AA	41	1.00	0.85 - 1.18	0.054
		AG	45	1.28	1.10 - 1.50	
		GG	17	1.16	0.91 - 1.46	
						
*NOTCH2NL*	chr1: 120,338,694 - 120,398-362	AA	41	1.00	0.75 - 1.33	0.038
	chr1: 143,920,468 - 144,009,723	AG	47	0.64	0.49 - 0.83	
		GG	19	0.70	0.47 - 1.04	
						
*FAM72B*	chr1: 120,640,935 - 120,657,204	AA	41	1.00	0.68 - 1.47	0.218
		AG	47	0.76	0.53 - 1.10	
		GG	19	0.59	0.35 - 1.00	
						
*SRGAP2*	chr1: 120,808,675 - 120,832,674	AA	41	1.00	0.82 - 1.21	0.652
	chr1: 204,582,823 - 204,648,014	AG	48	1.03	0.86 - 1.24	
		GG	19	0.89	0.68 - 1.17	
						
rs11249433	chr1: 120,982,136					

^+ ^Based on the UCSC genome browser on Human Mar. 2006 assembly (hg18).

*Generalized linear model adjusted for age, ER and PR status.

Exons 2 - 4 of *NOTCH2 *are included in *NOTCH2NL *gene, and this region is duplicated at 1q21.1, on the other side of centromere, while exons 5 - 34 of *NOTCH2 *are uniquely mapped to the 1p11.2 region. The full-length *NOTCH2 *has 34 exons that encode a protein of 2471 amino acids with an extracellular domain containing multiple epidermal growth factor (EGF)-like repeats, and *NOTCH2NL *has 4 exons and encodes a protein of 235 amino acids with only one EGF-like repeat. The role of *NOTCH2NL *is not clear and, given its mapping to multiple locations and the lack of protein domains, expression of *NOTCH2NL *was not studied further.

### *NOTCH2 *expression analysis

To corroborate our observations of the suggestive association between rs11249433 and *NOTCH2 *expression, we employed the sensitive and quantitative reverse-transcriptase PCR (RT-PCR) method in an extended set of 180 breast tumors, 104 of which were used for the initial microarray analysis. We used a custom-designed expression assay for terminal exons 33 and 34 of *NOTCH2 *to detect the full-length transcripts (*FL-NOTCH2*). Expression of *FL-NOTCH2 *was increased in tumors from individuals with risk genotypes AG and GG of rs11249433 compared to non-risk AA genotypes by 1.24 and 1.12 fold, respectively (p = 0.066, Additional File 1, Table S1). The expression was found to be significantly increased in tumors from patients with higher age at diagnosis (p = 0.0007) and decreased in tumors with *TP53 *mutations (p = 0.0056, Additional File 1, Table S1). However, no association was observed between *NOTCH2 *expression and tumor grade or stage (data not shown).

**Figure 2:**
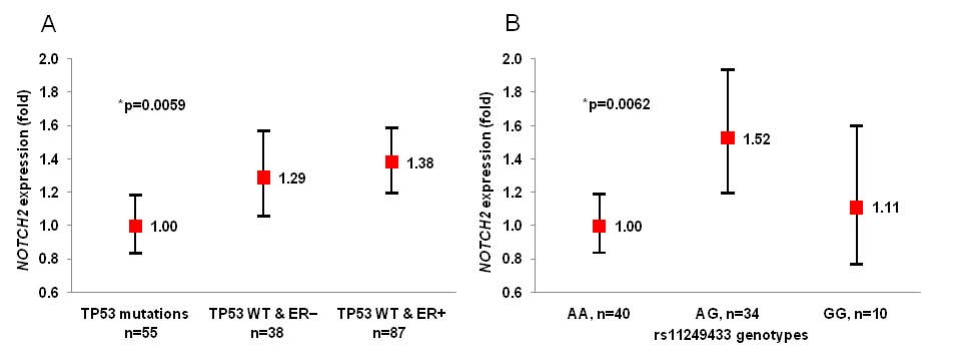
***NOTCH2 *expression in breast tumors**. A. Stratified by ER and *TP53 *mutational status; B. Stratified by genotypes of rs11249433 in *TP53wt*/ER^+ ^tumors. Expression is shown as fold difference with 95% confidence intervals compared to reference groups; p-values are for generalized linear model adjusted for plate effect, age at diagnosis, race, ER/PR and *TP53 *mutational status when applicable.

The tumor suppressor gene *TP53 *regulates multiple vital cell functions. Germline *TP53 *mutations found in patients with Li-Fraumeni syndrome [26] are associated with early development of tumors, while the restoration of the gene function causes regression of tumors by apoptosis and senescence [27]. Somatic mutations in the *TP53 *gene are found in 20 - 30% of all breast tumors [28,29], but the frequency varies by tumor stage and subtype. For example, more than 80% of basal-like tumors, while only 13% of ER^+ ^luminal tumors carry *TP53 *mutations [15]. Since different subtypes of BC have been associated with distinct gene expression profiles [12-14], we examined the *NOTCH2 *expression in subgroups of breast tumors according to their ER and *TP53 *status. There was a strong difference in *NOTCH2 *expression in these subgroups (p = 0.0059, Figure 2A and Additional File 1, Table S1), the expression was lower in tumors with *TP53 *mutations. In tumors with wild-type (wt) *TP53 *gene, *NOTCH2 *expression was higher in ER^+ ^compared to ER^- ^tumors. Among patients with *TP53wt*/ER^+ ^tumors, *NOTCH2 *expression was increased by 1.52 and 1.11 fold in individuals with the risk AG and GG genotypes, respectively, compared to those with the non-risk AA genotype of rs11249433 (p = 0.0062, Figure 2B and Additional File 1, Table S1). SNP rs11249433 is located ~600 kb away from *NOTCH2*. The non-linear increase of *NOTCH2 *expression with allele counts may indicate that rs11249433 is not the actual risk factor but is in LD with the best candidate that can be located at any distance.

There was no association of rs11249433 with *NOTCH2 *expression in RNA from peripheral blood of 302 women with a previous breast cancer diagnosis (Additional File 1, Table S2), or in 76 samples of adjacent normal breast tissue (Additional File 1, Table S3). According to GNF Expression Atlas 2 data http://biogps.gnf.org/#goto=genereport&id=4853 and results by Ohishi et al. [30], the highest *NOTCH2 *expression of all blood fractions is found in monocytes while it is low in all other blood cells. Using purified peripheral blood monocytes from 60 healthy controls, we observed that individuals carrying risk genotypes of rs11249433 had significant increase of *NOTCH2 *expression (p = 0.015, Additional File 1, Table S4), similar to the pattern in breast tumors.

The pericentromeric *NOTCH2 *region is reported to contain large structural variations (amplifications and deletions) detectable by high-resolution comparative genomic hybridization (CGH) analysis [31]. We hypothesized that these copy number variations (CNVs) might affect mRNA expression of *NOTCH2*, and measured CNVs by two quantitative PCR assays located in intron 1 and upstream from *NOTCH2*. We used DNA from breast tumors (n = 97) and blood samples from BC patients (n = 295) in which expression analysis was performed. *NOTCH2 *expression was not significantly correlated with CNVs in both samples (Additional File 1, Table S5). Inclusion of CNVs into the multivariable model did not affect the association between rs11249433 and *NOTCH2 *expression (Additional File 1, Table S6). Another SNP, rs10923931, located within intron 2 of *NOTCH2 *has been reported to be associated with risk of type 2 diabetes [32]. We did not observe any effect of rs10923931 on *NOTCH2 *expression in breast tumor tissue (Additional File 1, Table S1) or in blood of BC patients (Additional File 1, Table S2).

### Assessment of possible *NOTCH2 *function through expression and pathway analyses in NCI-60 cell lines

*NOTCH2 *belongs to a family of transmembrane receptors involved in regulation of cell-cell communication, cell proliferation, differentiation, and apoptosis [33]. However, the specific role of *NOTCH2 *is not yet well established. To assess the potential function of *NOTCH2*, we used expression data generated in 60 human cell lines representing several cancer types (NCI-60 cell line expression set [34]). We examined correlation patterns between *NOTCH2 *and all the genes represented on Affymetrix HG-U133 arrays, as previously described [35]. After adjustment for multiple comparisons, 469 and 758 genes showed significantly positive and negative correlations with *NOTCH2*, respectively. These genes were analyzed for enrichment of Gene Ontology (GO) categories. The analysis identified statistically significant enrichment of the integrin signaling pathway (p = 1.87 × 10^-7^) and cell structure and motility (p = 6.96 × 10^-9^) for genes with positive correlations with *NOTCH2*, and enrichment for nucleic acid binding (p = 1.88 × 10^-17^) and nucleoside, nucleotide and nucleic acid metabolism (p = 1.20 × 10^-10^) for genes with negative correlations with *NOTCH2 *(Table 2). These results supported the potential role of *NOTCH2 *in maintaining cell shape, structure and motility [36], and NOTCH-mediated regulation of integrin affinity [37].

**Table 2:** Summary of PANTHER pathway enrichment analysis for transcripts correlated with *NOTCH2 *in the NCI-60 set of cell lines

PANTHER category^a^		No. of genes in reference list	Observed no. of genes in category	Expected no. of genes in category	Over(+)/under(-) representation	p-value^b^
***For genes with positive correlation with NOTCH2 expression***						

BP00285	Cell structure and motility	990	65	27.7	+	6.96 × 10^-9^
P00034	Integrin signalling pathway	217	26	6.1	+	1.87 × 10^-7^
MF00091	Cytoskeletal protein	698	47	19.5	+	1.22 × 10^-6^
BP00125	Intracellular protein traffic	892	51	25.0	+	4.59 × 10^-5^
MF00261	Actin binding cytoskeletal protein	342	29	9.6	+	3.46 × 10^-5^

***For genes with negative correlation with NOTCH2 expression***						

MF00042	Nucleic acid binding	2214	187	100.1	+	1.88 × 10^-17^
BP00031	Nucleoside, nucleotide and nucleic acid metabolism	2837	203	128.3	+	1.20 × 10^-10^
BP00047	Pre-mRNA processing	264	35	11.9	+	4.74 × 10^-6^
BP00048	mRNA splicing	196	29	8.9	+	1.04 × 10^-5^

^a^PANTHER accession number for pathway (P), biological process (BP), or molecular function (MF) http://www.pantherdb.org/.

^b^p-values of binomial test with Bonferroni correction for multiple testing.

### Expression and functional characterization of splice variants of *NOTCH2*

**Figure 3:**
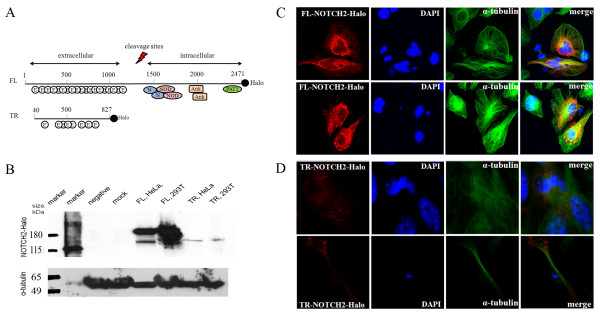
**Splicing variation of *NOTCH2***. A. Schematic representation of recombinant constructs for full-length (FL) and truncated (TR) NOTCH2 protein isoforms fused with C-terminal Halo-tag protein (marked as Halo). Intracellular part of FL-NOTCH2 consists of two LNRs (Lin-12/Notch repeat domains, marked as N), two NOTCH protein domains (marked as NOD), two ankyrin repeats (marked as ANK) and a topoisomerase II-associated protein domain (marked as PAT1). B. Western blot showing expression of recombinant FL-NOTCH2-Halo and TR-NOTCH2-Halo constructs in HeLa and 293T cell lines. The lanes show two different size markers, negative control (no DNA), mock control (vector only) and results of transfections with FL-NOTCH2-Halo and TR-NOTCH2-Halo in HeLa and 293T cells. C. Confocal imaging of protein expression for the recombinant construct FL-NOTCH2-Halo (red) in HeLa cells. Untransfected cells in the same field serve as negative controls. Antibodies against α-tubulin were used to stain the cytoskeleton (green) while DAPI was used to stain the cell nuclei (blue). D. Confocal imaging of protein expression for the recombinant construct TR-NOTCH2-Halo (red) in HeLa cells. Untransfected cells in the same field serve as negative controls. Antibodies against α-tubulin were used to stain the cytoskeleton (green) while DAPI was used to stain the cell nuclei (blue).

We tested for potential alternative splicing events within the *NOTCH2 *gene by performing a 5' rapid amplification of cDNA ends (RACE). One of the transcripts with alternative 5'end was identical to *NOTCH2NL *(data not shown). Another alternative splicing form was transcribed from an alternative exon 1a and was terminated by an alternative stop codon after exon 16 of the *NOTCH2 *gene (GenBank GQ231534). This splicing form encodes a truncated (TR) protein of 863 amino acids identical to a fragment of the full-length (FL) NOTCH2 protein (amino acids 40 - 827). The TR-NOTCH2 protein is predicted to have a dominant negative function relative to FL-NOTCH2, as it has an extracellular but no intracellular domain (Figure 3A). FL-NOTCH2 protein has 19 epidermal growth factor (EGF)-like repeats, while the TR-NOTCH2 has only 8 repeats, thus the functional properties of extracellular domains of these two protein forms and their interactions with ligands might be different.

We cloned both splicing forms of *NOTCH2 *and examined their protein expression in human cell lines HeLa and 293T. NOTCH proteins undergo several stages of proteolytic cleavages: non-activating cleavage at S1 site with furin-like convertase occurs during protein processing and generates two protein subunits - extracellular and transmembrane/intracellular; S2 activating cleavage occurs in response to ligand binding; S3 activating cleavage by γ-secretase generates the notch intracellular domain (NICD) that migrates to the nucleus and activates downstream targets [22]. The use of γ-secretase inhibitors that prevent NOTCH cleavage at S3 site has been targeted as a promising tool for anti-cancer therapy [38]. The expected sizes for FL-NOTCH2-Halo and TR-NOTCH2-Halo expression constructs with C-terminal Halo-tags are ~298 kDa and ~125 kDa, respectively. The results of the Western blot (Figure 3B) suggested that the observed size of ~200 kDa (with Halo-Tag of 33 kDa) for FL-NOTCH2-Halo is likely to correspond to C-terminal intracellular part of protein after non-activating S1 cleavage. The shorter bands might correspond to forms after activation cleavage events at S2 and S3 sites. For TR-NOTCH2-Halo that lacks the cleavage site, low protein expression was detected by a band of a size corresponding to the unprocessed protein (~125 kDa). Confocal imaging of both expression constructs showed a differential pattern of cellular localization for these forms. The expression pattern for FL-NOTCH2-Halo suggested that the C-terminal intracellular protein part is localized in the cytoplasm and in the peri-nuclear space (Figure 3C). In contrast, the TR-NOTCH2 form, which lacks the cleavage sites, is found in a granule-type pattern, possibly anchored to cell membrane (Figure 3D).

mRNA expression of *TR-NOTCH2 *was lower than that of *FL-NOTCH2 *by 10.7 fold in blood samples, by 25.4 fold in tumor breast tissue and by 33.1 fold in normal breast tissue (Additional File 1, Figure S1). There was also a great variation in relative expression of *FL-NOTCH2 *and *TR-NOTCH2 *in a panel of human tissues and NCI-60 cancer cell lines, but the significance of this differential expression is unknown (Additional File 1, Table S7). The pattern of *NOTCH2 *mRNA expression in different tumor types and genotype groups was similar for both forms (Additional File 1, Table S1, Table S2, and Table S8), but due to a much lower level of expression of the *TR-NOTCH2 *form and a higher variability of this expression, the results were mostly not statistically significant (Additional File 1, Table S8). Therefore, the association between rs11249433 and expression of *NOTCH2 *in human breast tumors is likely to be general and unspecific for particular splicing forms. However, as a result of a much higher expression level of *FL-NOTCH2 *compared to *TR-NOTCH2 *in breast tissue, the effect of *FL-NOTCH2 *might be predominant.

## Conclusions

Large-scale GWAS for BC identified multiple genomic regions that harbor genetic risk factors for the disease. In this study, we aimed to identify a molecular phenotype related to the recently reported genetic association within the 1p11.2 region [4]. We found that the BC-associated rs11249433 located within this region, is associated with mRNA expression of *NOTCH2 *gene. Most unexpectedly, the association between rs11249433 and *NOTCH2 *expression was dependent on the mutational status of the tumor suppressor gene *TP53 *and ER status of the tumors. Among other scenarios this suggests that either the estrogen receptor or the *TP53 *may have a function in the regulation of *NOTCH2 *expression, as the restoration of p53 expression has been shown to affect *NOTCH1 *expression [39,40]. An active NOTCH pathway is important for the induction of breast stem cells to differentiate into luminal cells of breast ducts [41]. Thus, increased or persistent activation of *NOTCH2 *expression may favor development of ER^+ ^breast carcinomas in *TP53wt *tumors. Indeed, the original GWAS association with rs11249433 was stronger in ER^+ ^compared to ER^- ^cancer [4]. Future research will have to examine how rs11249433 influences *NOTCH2 *expression and what function *TP53 *and the estrogen receptor may have in regulation of *NOTCH2 *expression. We also identified the first possible dominant-negative truncated form of NOTCH2. The exact role of this form in different tissues and biological conditions warrants further studies.

## Materials and methods

### Human breast tissue and blood samples

Fresh-frozen tumor specimens and blood samples were obtained from breast cancer patients. The US breast tissue samples (100 tumor samples and 90 adjacent normal breast tissue) were previously described [42]. Briefly, patients were recruited at the University of Maryland Medical Center (UMD), the Baltimore Veterans Affairs Medical Center, Union Memorial Hospital, Mercy Medical Center, and the Sinai Hospital in Baltimore between 1993 and 2003. All patients signed a consent form and completed an interviewer-administered questionnaire. Clinical and pathological information was obtained from medical records and pathology reports. Disease staging was performed according to the tumor-node-metastasis (TNM) system of the American Joint Committee on Cancer/the Union Internationale Contre le Cancer (AJCC/UICC). The Nottingham system was used to determine the tumor grade. The collection of tumor specimens, survey data, and clinical and pathological information was reviewed and approved by the University of Maryland Institutional Review Board for the participating institutions (UMD protocol #0298229). IRB approval of this protocol was then obtained at all institutions (Veterans Affairs Medical Center, Union Memorial Hospital, Mercy Medical Center, and Sinai Hospital). The project was also reviewed and approved by the NIH Office of Human Subjects Research (OHSR #2248).

Breast tumor samples from Norway (n = 116) were collected in 1995-1998 from 5 hospitals located in the Oslo region [43]. Histological tumor type, tumor size, and nodal involvement were analyzed, and the disease was staged according to the TNM system. Tumor grading was performed according to Elston and Ellis [44] and tumor slides were screened for vascular invasion. Blood samples (n = 316) were collected from breast cancer survivors treated with radiotherapy at the Norwegian Radium Hospital between 1998 and 2002 and invited in 2004 to participate in a study assessing late clinical and biochemical effects [45,46]. The project was reviewed and approved by the NIH Office of Human Subjects Research (OHSR #4700). Monocytes from fresh blood obtained from anonymous blood donors at the NIH Blood Bank, were described previously [47].

Overall, all blood samples were collected from patients of European ancestry, while for breast tissue 75% samples were from patients of European ancestry and 25% of African ancestry. Race of patients was included as a covariate in the generalized linear model when relevant.

### ER, PR status evaluation

For the US samples, the ER status was determined immunohistochemically using formalin-fixed, paraffin-embedded tissue slides and the ready-to-use monoclonal antibody (clone 6F11, Ventana Medical Systems, Tucson, AZ). The ER status was determined according to the reference range set by the ChromaVision ACIS assisted quantitative image analysis software (Clarient Diagnostic Services, Irvine, CA) and was scored as negative/positive. The PR status was abstracted from medical records, as available. For the Norwegian samples, immunostaining was performed using mouse mAb against estrogen receptor (ER) and progesterone receptor (PgR; clones 6F11 and 1A6, respectively; Novocastra, Newcastle on Tyne, United Kingdom). Automated immunostaining systems BioGenex were used for both markers. Immunopositivity was recorded for tumors with > 10% (ER, PgR) of staining.

### *TP53 *mutation analysis

Tumors were screened for *TP53 *mutations as previously described [43,48].

### RNA preparation

Total RNA from fresh-frozen breast tissue samples was isolated using TRIZOL reagent according to the manufacturer's instructions (Invitrogen, Carlsbad, CA). Total RNA from blood cells was isolated from PAX tubes at AROS Applied Biotechnology (Aarhus, Denmark) with an automated procedure utilizing the column-based technologies from Qiagen Inc. (Hilden, Germany). The concentration was measured using a NanoDrop spectrophotometer (NanoDrop Technologies, Wilmington, DE). RNA integrity for each sample was confirmed with the Agilent 2100 Bioanalyzer (Agilent Technologies, Palo Alto, CA).

### cDNA preparation

For each sample, 200-300 ng of total RNA was converted to cDNA with Superscript III kit (Invitrogen) for the US samples or with High-Capacity cDNA Reverse Transcription Kit (Applied Biosystems, Foster City, CA) for the Norwegian samples.

### Microarray expression of 5 genes with Agilent 44K whole-genome microarrays

mRNA expression values for *ADAM30*, *NOTCH2*, *NOTCH2NL*, *FAM72B*, and *SRGAP2 *were extracted from a whole-genome expression set for breast tumor samples from Norway (unpublished data Edvardsen, et al.) generated with Agilent whole human genome 44K 60-mer oligo arrays, Agilent Technologies). The arrays were scanned using the Agilent scanner G2565A and signals were extracted using Feature Extraction v9.5. Data were log2 transformed and, after removal of non-uniform spots and exclusion of population outliers, replicated probes were averaged. The data were quintile normalized using the R package "normalize Between Arrays" from the LIMMA library [49] and missing values imputed using LLS imputation [50]. Information on genotypes, age, ER and PR status was available for 108 breast cancer samples with expression values.

### Genotyping

Pre-developed TaqMan allelic discrimination genotyping assays for SNPs rs11249433 (C___31617470_30) and rs10923931 (C___1188816_10) were purchased from Applied Biosystems. Genotyping was performed in 384-well optical plates on the ABI PRISM 7900HT Sequence Detection System (Applied Biosystems) with 10 ng of genomic non-tumor DNA, 0.125 μL of 40 × TaqMan SNP genotyping assay, 2.5 μL of 2 × TaqMan genotyping master mix (Applied Biosystems) in 5 μL reaction volume.

### Quantitative Reverse-Transcriptase PCR (qRT-PCR)

Expression of *NOTCH2 *was measured with two custom-designed assays, *FL-NOTCH2*, targeting a junction between the most terminal exons 33 and 34 and *TR-NOTCH2 *for the truncated form (information about all assays is presented in Additional File 1, Table S6). Three endogenous controls, glyceraldehyde-3-phosphate dehydrogenase (*GAPDH*), beta-2-microglobulin (*B2 M*) and cyclophilin (*PPIA*), were selected as appropriate controls based on prior expression studies in breast cancer [51]. All assays were validated on a cDNA panel prepared from a normal breast tissue sample at 8 different RNA concentrations: 1000 ng/μL, 100 ng/μL, 10 ng/μL, 1 ng/μL, 0.1 ng/μL, 0.01 ng/μL, 0.001 ng/μL, and 0 ng/μL. Standard curves constructed for each assay were used for calculation of amplification efficiency (E, %). To determine optimal amount of cDNA to be used for each assay, several samples representing each tissue type were tested at three 5-fold dilutions of cDNA. We used 2.4 ng of total RNA/reaction for *TR-NOTCH2 *assay and 0.6 ng of total RNA/reaction for all other assays. All plates included two negative controls (genomic DNA and water), and three positive controls prepared from total RNA of MCF7 breast cancer cell line and used at three 5-fold dilutions of cDNA. The positive controls were also used as interplate controls to normalize results between plates. All assays were run in quadruplicates on 384-well plates on ABI PRISM 7900HT Sequence Detection System (Applied Biosystems). For endogenous controls, the reactions were performed with cDNA prepared from 0.6 ng of total RNA, 0.25 μL of 20 × TaqMan expression assay and 2.5 μL of 2 × TaqMan Gene Expression master mix (Applied Biosystems) in 5 μL reaction volume. For *NOTCH2 *assays, the reactions were performed with cDNA prepared from 0.6 or 2.4 ng of total RNA, custom-designed primers, 2.5 μL of 2 × Power SYBR master mix (Applied Biosystems) in 5 μL reaction volume.

### CNV analysis

Two assays were used to quantify CNV around *NOTCH2 *region (Additional File 1, Table S9 for assay information). A CNV assay BC1 was designed to map upstream *NOTCH2 *and a CNV assay BC2 located within intron 1 of *NOTCH2*, was designed next to an assay that was previously used to validate genomic amplification of this region (Supplementary Table 4 of [31], Chr1tp-38C8, chr1: 120,245,520-120,395,759). Each of assays was quantified with qPCR using 5 ng of genomic DNA from corresponding samples (blood or tumor) and 2 × Power SYBR master mix (Applied Biosystems). All plates included two negative controls (water), and three positive controls (20 ng, 4 ng and 0.8 ng of a normal DNA sample). The positive controls were also used as interplate controls to normalize results between plates. All assays were run in quadruplicates on 384-well plates on ABI PRISM 7900HT Sequence Detection System (Applied Biosystems). The CNV assays were normalized by a control RNAseP assay (Applied Biosystems) run in quadruplicates in separate reactions.

### Pathway analysis

Expression data of NCI-60 cell lines on Affymetrix HG-U133 arrays that included 39,135 probes covering 18,457 unique annotated transcripts was downloaded from CellMiner Build1.0 [34]. The probe (212377_s_at) residing on the junction of exons 33 and 34 of *NOTCH2 *(similarly to *FL-NOTCH2 *expression assay) was selected to calculate the pairwise Pearson's correlation coefficient between this probe and all other probes on HG-U133 chip across the entire NCI-60 cancer cell panel. The false discovery rate (FDR) [52] was set at 5%, defining the threshold for correlation coefficient as r = +/- 0.39. The lists of genes that showed significantly positive or negative correlations with *NOTCH2 *expression were used to determine enrichment for any pathway, biological process, or molecular function using the *Compare Gene Lists Tool *from the PANTHER Classification System [53] relative to the reference gene list that included all 39,135 probes represented on Affymetrix HG-U133 arrays. Genes that showed positive and negative association with *NOTCH2 *were examined separately and the Bonferroni correction was applied for multiple testing.

### Statistical analyses

Expression analysis was performed as follows: for each assay, the mean values of quadruplicates were recalculated based on standard curve slopes to correspond to E = 100% of amplification efficiency as E = -1+10^(-1/slope) ^http://www.stratagene.com/techtoolbox/calc/qpcr_slope_eff.aspx. Distribution of expression values for each of the assays was tested for normality and no significant deviations were observed. The values for all assays were normalized to interplate controls on each plate. The expression values for *NOTCH2 *assays were normalized by the geometric mean of all endogenous controls (*GAPDH, B2 M *and *PPIA) *according to relative quantification method (dCt) [54]. A generalized linear model adjusted for applicable covariates (plate effect, age at diagnosis, race, ER/PR and *TP53 *mutation status) was used to test the association between mRNA expression and SNPs. For correlation analysis, Pearson's correlation coefficients and FDR-adjusted p-values were calculated. All the statistical analyses were performed using SAS/STAT system version 9.2 (SAS Institute Inc., Cary, NC).

### *NOTCH2 *cDNA cloning, transfection, Western blot and confocal imaging

5'RACE was performed starting with a mix of total RNA from normal and cancer breast tissue. The 5'RACE cDNA was prepared with SMART RACE cDNA Amplification Kit (Clontech, Mountain View, CA). Using the 5'RACE cDNA as a template, PCR fragments were amplified with a primer for exon 4 of *NOTCH2 *and a universal RACE linker attached to 5'end of transcripts. After sequencing and analysis of the PCR fragments, a novel alternative transcription start and exon 1a were identified. Using primers for two transcription starts and two translation stops predicted in the UCSC browser, all four combinations of PCR reactions were attempted on a breast tumor and a normal sample (primers are presented in Additional File 1, Table S9). The successful PCR products were cloned into a pFC8A Halo-tag CMV Flexi vector (Promega, Madison, WI) and multiple clones were sequenced. Only two splicing forms were identified - a known form (NM_024408), representing the full-length *FL-NOTCH2 *and a novel splicing form, *TR-NOTCH2 *(GenBank GQ231534). Equal amounts of the constructs were transfected into human HeLa and 293T cell using Lipofectamine 2000 reagent according to instructions (Invitrogen). Non-transfected cells (Lipofectamine only) and cells transfected with empty GFP-tagged pIRES vector (Clontech) served as negative controls. Protein lysates from transfected cells were prepared 48 hours post-transfection and analyzed by Western blot. Similar amounts of protein for each sample were resolved on 4-12% tris-glycine polyacrylamide gel (Invitrogen) together with pre-stained Rainbow marker (BenchMark Prestained Protein Ladder, Invitrogen) and Santa-Cruz protein marker (sc-2035, Cruz Marker Molecular Weight Standards, Santa Cruz, CA), transferred to nitrocellulose membrane (Invitrogen), blocked with 5% milk and probed with rabbit anti-Halo (Promega) and mouse α-tubulin primary antibodies (ab-7291, Abcam, Cambridge, MA), both at 1:1000 dilution, followed by incubation with secondary HRP-labeled antibodies (sc-2305 donkey anti-rabbit and sc-2302 goat anti-mouse, Santa Cruz, at 1:10,000 dilution) and ECL-detection (GE Healthcare, Pittsburgh, PA). For immunofluorescence analysis, the cells were grown on chamber slides and transfected with the expression constructs with Lipofectamine 2000 reagent according to instructions (Invitrogen). 48 hours post-transfection, the cells were permeabilized with 4% of paraformaldehyde, blocked with 4% BSA in TBS for 1 hr, and incubated with primary rabbit Anti-Halotag antibody (Promega), and mouse α-tubulin (ab-7291, Abcam), both at 1:1000 dilution. Cells were incubated with secondary antibody (donkey anti-rabbit Alexa Fluor 594 and donkey anti-mouse Alexa Fluor 488, Invitrogen, both at 1:10,000 dilution) for 1 hr. Slides were covered with mounting media (Prolong Gold antifade reagent with DAPI, Invitrogen) and images were collected with a confocal laser scanning microscope (LSM 510 META, Carl Zeiss Jena GmbH, Jena, Germany) at ×63 magnification with oil.

### Bioinformatics analysis of protein domains

Sequences for *NOTCH2 *splicing forms were analyzed for presence of open reading frames http://www.ncbi.nlm.nih.gov/projects/gorf/ and putative conserved domains http://www.ncbi.nlm.nih.gov/Structure/cdd/wrpsb.cgi. Molecular weight of protein isoforms was calculated with Protein Calculator v3.3 http://www.scripps.edu/~cdputnam/protcalc.html.

## List of Abbreviations

GWAS: genome-wide association study; SNP: single nucleotide polymorphism; BC: breast cancer; LD: linkage disequilibrium; ER: estrogen receptor; PR: progesterone receptor; HER-2: human epidermal growth factor receptor 2.

## Competing interests

The authors declare that they have no competing interests.

## Authors' contributions

YPF, HE and LPO designed the study; HLH, SDF, SA, BN, ALBD and VNK provided samples; YPF, HE, AK, JPA, TMH, IK, PPG, AS performed the study; YPF, HE and LPO wrote the paper. All authors approved the final version of the manuscript and its submission for publication.

## Supplementary Material

Additional file 1 **Table S1**. Expression of *FL-NOTCH2 *in breast tumor samples. **Table S2**. Expression of *FL-NOTCH2 *in blood samples from breast cancer patients. **Table S3**. Expression of *FL-NOTCH2 *and *TR-NOTCH2 *in normal breast samples. **Table S4**. Expression of *FL*-*NOTCH2 *in purified peripheral blood monocytes from healthy controls. **Table S5**. Pearson correlation coefficient between *NOTCH2 *expression and copy number variation. **Table S6**. Test for effect of CNVs on association of rs11249433 with *NOTCH2 *expression. **Table S7**. Fold difference in expression of *FL-NOTCH2 *compared to *TR-NOTCH2 *in NCI-60 cancer cell lines and a panel of normal and tumor human tissues. **Table S8**. Expression of *TR-NOTCH2 *in breast tumor and blood samples. **Table S9**. List of primers and assays. **Figure S1**. Expression of two splicing forms of *NOTCH2 *in blood, tumor and normal breast tissue samples. Click here for file
