# Supplementary material for: NOTCH2 in breast cancer: association of SNP rs11249433 with gene expression in ER-positive breast tumors without TP53 mutations
Source: Mol Cancer. 2010 May 19;9:113. doi: 10.1186/1476-4598-9-113 (PMC2887795; doi:10.1186/1476-4598-9-113)
Supplement: Additional file 1 — Table S1. Expression of FL-NOTCH2 in breast tumor samples. Table S2. Expression of FL-NOTCH2 in blood samples from breast cancer patients. Table S3. Expression of FL-NOTCH2 and TR-NOTCH2 in normal breast samples. Table S4. Expression of FL-NOTCH2 in purified peripheral blood monocytes from healthy controls. Table S5. Pearson correlation coefficient between NOTCH2 expression and copy number variation. Table S6. Test for effect of CNVs on association of rs11249433 with NOTCH2 expression. Table S7. Fold difference in expression of FL-NOTCH2 compared to TR-NOTCH2 in NCI-60 cancer cell lines and a panel of normal and tumor human tissues. Table S8. Expression of TR-NOTCH2 in breast tumor and blood samples. Table S9. List of primers and assays. Figure S1. Expression of two splicing forms of NOTCH2 in blood, tumor and normal breast tissue samples. [file 1476-4598-9-113-S1.DOC]

**ADDITIONAL FILES**

**Table S1.** Expression of *FL-NOTCH2* in breast tumor samples.

| **Variable** | **N** | **Est. fold*** | **95%CI** | **p-value*** | **Covariates** | **p-value*** |
| --- | --- | --- | --- | --- | --- | --- |
| ***All study subjects*** | | | | | | |
| rs11249433 AA | 87 | 1.00 | 0.88 - 1.13 | 0.066 | Plate effect | <0.001 |
| AG | 67 | 1.24 | 1.06 - 1.46 |  | Age at diagnosis | 0.0007 |
| GG | 23 | 1.12 | 0.87 - 1.43 |  | Race | 0.475 |
|  |  |  |  |  | ER status | 0.401 |
|  |  |  |  |  | PR status | 0.122 |
|  |  |  |  |  | *TP53* mutations | 0.0056 |
| rs10923931 GG | 91 | 1.00 | 0.89-1.14 | 0.679 | Plate effect | <0.001 |
| GT | 23 | 0.95 | 0.75-1.20 |  | Age at diagnosis | 0.003 |
| TT | 0 | -- | -- |  | ER status | 0.957 |
|  |  |  |  |  | PR status | 0.331 |
|  |  |  |  |  | *TP53* mutations | 0.015 |
| *TP53* mutations | 55 | 1.00 | 0.85 - 1.18 | 0.0059 | Plate effect | <0.001 |
| *TP53*wt/ER^−^ | 38 | 1.29 | 1.06 - 1.57 |  | Age at diagnosis | 0.002 |
| *TP53*wt/ER^+^ | 87 | 1.38 | 1.20 - 1.59 |  | Race | 0.190 |
|  |  |  |  |  | PR status | 0.141 |
| ***Individuals with TP53wt/ER^+^ tumors*** | | | | | | |
| rs11249433 AA | 40 | 1.00 | 0.84 - 1.19 | 0.0062 | Plate effect | <0.001 |
| AG | 34 | 1.52 | 1.20 - 1.94 |  | Age at diagnosis | 0.001 |
| GG | 10 | 1.11 | 0.77 - 1.60 |  | Race | 0.611 |
|  |  |  |  |  | PR status | 0.587 |
| ***Individuals with TP53wt/ER^−^ tumors*** | | | | | | |
| rs11249433 AA | 18 | 1.00 | 0.63-1.59 | 0.458 | Plate effect | 0.124 |
| AG | 14 | 0.98 | 0.64-1.48 |  | Age at diagnosis | 0.684 |
| GG | 6 | 1.41 | 0.78-2.52 |  | Race | 0.978 |
|  |  |  |  |  | PR status | 0.489 |
| ***Individuals withTP53 mutations*** | | | | | | |
| rs11249433 AA | 29 | 1.00 | 0.78-1.28 | 0.947 | Plate effect | <0.001 |
| AG | 19 | 1.01 | 0.76-1.43 |  | Age at diagnosis | 0.018 |
| GG | 7 | 1.07 | 0.67-1.72 |  | Race | 0.290 |
|  |  |  |  |  | ER status | 0.735 |
|  |  |  |  |  | PR status | 0.052 |

*Generalized linear model adjusted for covariates.

**Table S2. Expression of *FL-NOTCH2* in blood samples from breast cancer patients.**

| **Variable** | **N** | **Est. fold*** | **95%CI** | **p-value*** | **Covariates** | **p-value*** |
| --- | --- | --- | --- | --- | --- | --- |
| rs11249433 AA | 100 | 1.00 | 0.87-1.15 | 0.083 | Plate effect | <0.001 |
| AG | 128 | 0.94 | 0.81-1.09 |  | Age at diagnosis | 0.141 |
| GG | 61 | 0.88 | 0.75-1.04 |  |  |  |
| rs10923931 GG | 232 | 1.00 | 0.95-1.06 | 0.904 | Plate effect | <0.001 |
| GT | 67 | 0.98 | 0.90-1.07 |  | Age at diagnosis | 0.116 |
| TT | 3 | 0.96 | 0.65-1.41 |  |  |  |

*****Generalized linear model adjusted for covariates.

**Table S3.** Expression of *FL-NOTCH2* and *TR-NOTCH2* in normal breast samples.

| **Variable** | **N** | **Est. fold*** | **95%CI** | **p-value*** | **Covariates** | **p-value*** |
| --- | --- | --- | --- | --- | --- | --- |
| ***FL-NOTCH2*** | | | | | | |
| rs11249433 AA | 49 | 1.00 | 0.86 - 1.17 | 0.381 | Plate effect | 0.104 |
| AG | 21 | 1.09 | 0.87 - 1.36 |  | Age at diagnosis | 0.307 |
| GG | 6 | 1.35 | 0.91 - 2.01 |  | Race | 0.494 |
| ***TR-NOTCH2*** | | | | | | |
| rs11249433 AA | 48 | 1.00 | 0.62 - 1.63 | 0.522 | Plate effect | 0.218 |
| AG | 20 | 0.85 | 0.42 - 1.73 |  | Age at diagnosis | 0.687 |
| GG | 6 | 1.84 | 0.54 - 6.29 |  | Race | 0.229 |

*****Generalized linear model adjusted for covariates.

**Table S4.** Expression of *FL***-***NOTCH2* in purified peripheral blood monocytes from healthy controls.

| **Variable** | **N** | **Est. fold*** | **95%CI** | **p-value*** | **Covariates** | **p-value*** |
| --- | --- | --- | --- | --- | --- | --- |
| ***FL-NOTCH2*** | | | | | | |
| rs11249433 AA | 27 | 1.00 | 0.90 - 1.11 | 0.015 | Age at diagnosis | 0.067 |
| AG | 26 | 1.11 | 1.00 - 1.22 |  |  |  |
| GG | 7 | 1.33 | 1.11 - 1.58 |  |  |  |

*Generalized linear model adjusted for covariates.

**Table S5.** Pearson correlation coefficient between *NOTCH2* expression and copy number variation.

| **Sample source** | **n** | **CNV** | **r** | **p-value** |
| --- | --- | --- | --- | --- |
| Breast tumor tissue | 97 | BC1 | 0.185 | 0.070 |
|  | 97 | BC2 | 0.095 | 0.355 |
| Blood | 295 | BC1 | 0.107 | 0.069 |
|  | 295 | BC2 | 0.054 | 0.356 |

**Table S6.** Test for effect of CNVs on association of rs11249433 with *NOTCH2* expression.

| **rs11249433** | **No.** | **Est. fold*** | **95%CI** | **p-value*** | **Covariates** | **p-value*** |
| --- | --- | --- | --- | --- | --- | --- |
| ***In a subset of breast tumors with CNV data*** | | | | | | |
| AA | 34 | 1.000 | 0.832 - 1.202 | 0.192 | Plate effect | <0.001 |
| AG | 38 | 1.259 | 1.049 - 1.509 |  | Age at diagnosis | 0.020 |
| GG | 15 | 1.052 | 0.802 - 1.380 |  | ER status | 0.521 |
|  |  |  |  |  | PR status | 0.150 |
|  |  |  |  |  | TP53 mutation | 0.078 |
|  |  |  |  |  |  |  |
| AA | 34 | 1.000 | 0.831 - 1.203 | 0.199 | Plate effect | <0.001 |
| AG | 38 | 1.257 | 1.047 - 1.509 |  | Age at diagnosis | 0.025 |
| GG | 15 | 1.049 | 0.798 - 1.379 |  | ER status | 0.509 |
|  |  |  |  |  | PR status | 0.143 |
|  |  |  |  |  | TP53 mutation | 0.079 |
|  |  |  |  |  | **CNV-BC1** | 0.752 |
|  |  |  |  |  |  |  |
| AA | 34 | 1.763 | 1.496 - 2.030 | 0.197 | Plate effect | <0.001 |
| AG | 38 | 2.094 | 1.831 - 2.358 |  | Age at diagnosis | 0.023 |
| GG | 15 | 1.837 | 1.443 - 2.231 |  | ER status | 0.520 |
|  |  |  |  |  | PR status | 0.149 |
|  |  |  |  |  | TP53 mutation | 0.081 |
|  |  |  |  |  | **CNV-BC2** | 0.817 |
| ***In a subset of blood samples with CNV data*** | | | | | | |
| AA | 97 | 1.000 | 0.931 - 1.074 | 0.273 | Plate effect | <0.001 |
| AG | 126 | 0.959 | 0.898 - 1.025 |  | Age at diagnosis | 0.370 |
| GG | 60 | 0.921 | 0.846 - 1.002 |  |  |  |
|  |  |  |  |  |  |  |
| AA | 97 | 1.000 | 0.931 - 1.075 | 0.267 | Plate effect | <0.001 |
| AG | 126 | 0.957 | 0.896 - 1.023 |  | Age at diagnosis | 0.390 |
| GG | 60 | 0.921 | 0.846 - 1.002 |  | **CNV-BC1** | 0.582 |
|  |  |  |  |  |  |  |
| AA | 97 | 1.000 | 0.931 - 1.074 | 0.274 | Plate effect | <0.001 |
| AG | 126 | 0.959 | 0.897 - 1.025 |  | Age at diagnosis | 0.373 |
| GG | 60 | 0.921 | 0.846 - 1.002 |  | **CNV-BC2** | 0.934 |

*Generalized linear model adjusted for covariates.

**Table S7.** Fold difference in expression of *FL-NOTCH2* compared to *TR-NOTCH2* in NCI-60 cancer cell lines and a panel of normal and tumor human tissues.

| **Cell line** | **Tissue of origin** | **Source** | **Fold diff.*** | **Cell line** | **Tissue of origin** | **Source** | **Fold diff.*** |
| --- | --- | --- | --- | --- | --- | --- | --- |
| BT_549 | Breast | NCI-60 | 250 | SK_MEL_5 | Melanoma | NCI-60 | 76 |
| HS578T | Breast | NCI-60 | 86 | UACC_257 | Melanoma | NCI-60 | 74 |
| MCF7 | Breast | NCI-60 | 38 | UACC_62 | Melanoma | NCI-60 | 175 |
| MDA_MB_231 | Breast | NCI-60 | 76 | NCI_ADR_RES | Ovary | NCI-60 | 82 |
| T47D | Breast | NCI-60 | 104 | OVCAR_3 | Ovary | NCI-60 | 177 |
| SF_268 | CNS | NCI-60 | 61 | OVCAR_4 | Ovary | NCI-60 | 109 |
| SF_295 | CNS | NCI-60 | 236 | OVCAR_5 | Ovary | NCI-60 | 117 |
| SF_539 | CNS | NCI-60 | 93 | OVCAR_8 | Ovary | NCI-60 | 145 |
| SNB_19 | CNS | NCI-60 | 260 | SK_OV_3 | Ovary | NCI-60 | 122 |
| SNB_75 | CNS | NCI-60 | 269 | DU_145 | Prostate | NCI-60 | 77 |
| U251 | CNS | NCI-60 | 158 | PC_3 | Prostate | NCI-60 | 103 |
| COLO205 | Colon | NCI-60 | 23 | 786_0 | Kidney | NCI-60 | 80 |
| HCC_2998 | Colon | NCI-60 | 93 | ACHN | Kidney | NCI-60 | 145 |
| HCT_116 | Colon | NCI-60 | 275 | CAKI_1 | Kidney | NCI-60 | 134 |
| HCT_15 | Colon | NCI-60 | 84 | RXF_393 | Kidney | NCI-60 | 75 |
| HT29 | Colon | NCI-60 | 113 | SN12C | Kidney | NCI-60 | 100 |
| KM12 | Colon | NCI-60 | 58 | TK_10 | Kidney | NCI-60 | 110 |
| SW_620 | Colon | NCI-60 | 83 | UO_31 | Kidney | NCI-60 | 155 |
| A549 | Non-Small Cell Lung | NCI-60 | 209 | -- | Colon Tumor | Tumor tissue | 84 |
| EKVX | Non-Small Cell Lung | NCI-60 | 72 | -- | Kidney Tumor | Tumor tissue | 51 |
| HOP_62 | Non-Small Cell Lung | NCI-60 | 120 | -- | Lung Tumor | Tumor tissue | 119 |
| HOP_92 | Non-Small Cell Lung | NCI-60 | 95 | -- | Ovary Tumor | Tumor tissue | 84 |
| NCI_H226 | Non-Small Cell Lung | NCI-60 | 109 | -- | Thyroid Tumor | Tumor tissue | 28 |
| NCI_H23 | Non-Small Cell Lung | NCI-60 | 388 | -- | Adrenal | Normal tissue | 144 |
| NCI_H322M | Non-Small Cell Lung | NCI-60 | 136 | -- | Bladder | Normal tissue | 55 |
| NCI_H460 | Non-Small Cell Lung | NCI-60 | 59 | -- | Brain | Normal tissue | 31 |
| NCI_H522 | Non-Small Cell Lung | NCI-60 | 130 | -- | Breast | Normal tissue | 49 |
| CCRF_CEM | Leukemia | NCI-60 | 70 | -- | Colon | Normal tissue | 20 |
| HL_60 | Leukemia | NCI-60 | 41 | -- | Heart | Normal tissue | 22 |
| MOLT_4 | Leukemia | NCI-60 | 71 | -- | Kidney | Normal tissue | 39 |
| RPMI_8226 | Leukemia | NCI-60 | 128 | -- | Liver | Normal tissue | 54 |
| SR | Leukemia | NCI-60 | 66 | -- | Lung | Normal tissue | 22 |
| LOXIMVI | Melanoma | NCI-60 | 76 | -- | Muscle | Normal tissue | 44 |
| M14 | Melanoma | NCI-60 | 148 | -- | Pancreas | Normal tissue | 18 |
| MALME_3M | Melanoma | NCI-60 | 144 | -- | Prostate | Normal tissue | 46 |
| MDA_MB_435 | Melanoma | NCI-60 | 72 | -- | Small Intestine | Normal tissue | 25 |
| SK_MEL_2 | Melanoma | NCI-60 | 213 | -- | Spleen | Normal tissue | 26 |
| SK_MEL_28 | Melanoma | NCI-60 | 151 | -- | Stomach | Normal tissue | 71 |

*Expression is shown in fold difference between *FL-NOTCH2* compared to *TR-NOTCH2* splicing forms. Each tissue type is represented by one sample.

**Table S8.** Expression of *TR-NOTCH2* in breast tumor and blood samples.

| **Variable** | **N** | **Est. fold*** | **95%CI** | **p-value*** | **Adjustment** | **p-value*** |
| --- | --- | --- | --- | --- | --- | --- |
| ***In breast tissue samples*** | | | | | | |
| *TP53* mutations | 54 | 1.00 | 0.71 - 1.42 | 0.292 | Plate effect | 0.004 |
| *TP53*WT & ER− | 38 | 1.41 | 0.95 - 2.12 |  | Age at diagnosis | 0.072 |
| *TP53*WT & ER+ | 85 | 1.29 | 0.97 - 1.72 |  | Race | 0.826 |
|  |  |  |  |  | PR status | 0.292 |
| rs11249433 AA | 86 | 1.00 | 0.77 - 1.30 | 0.010 | Plate effect | 0.004 |
| AG | 65 | 1.80 | 1.28 - 2.54 |  | Age at diagnosis | 0.039 |
| GG | 23 | 1.46 | 0.87 - 2.45 |  | Race | 0.567 |
|  |  |  |  |  | ER status | 0.566 |
|  |  |  |  |  | PR status | 0.149 |
|  |  |  |  |  | *TP53* mutations | 0.290 |
| rs10923931 GG | 83 | 1.00 | 0.84-1.18 | 0.974 | Plate effect | <0.001 |
| GT | 21 | 1.01 | 0.73-1.39 |  | Age at diagnosis | 0.017 |
| TT | -- | -- | -- |  | ER status | 0.810 |
|  |  |  |  |  | PR status | 0.998 |
|  |  |  |  |  | *TP53* mutations | 0.009 |
| ***In blood samples*** | | | | | | |
| rs11249433 AA | 100 | 1.00 | 0.84 - 1.19 | 0.613 | Plate effect | <0.001 |
| AG | 128 | 0.99 | 0.83 - 1.18 |  | Age at diagnosis | 0.770 |
| GG | 61 | 0.94 | 0.78 - 1.14 |  |  |  |
| rs10923931 GG | 232 | 1.00 | 0.94 - 1.07 | 0.755 | Plate effect | <0.001 |
| GT | 67 | 1.04 | 0.94 - 1.16 |  | Age at diagnosis | 0.895 |
| TT | 3 | 0.98 | 0.63 - 1.54 |  |  |  |

*Generalized linear model adjusted for covariates.

**Table S9. List of primers and assays.**

| **Assay, primer set** | **Assay ID (ABI) or primer sequences** | **Assay type, cDNA amplicons size** |
| --- | --- | --- |
| **Endogenous controls:** |  |  |
| **Cyclophilin (*PPIA*)** | 4326316E | TaqMan |
| **Glyceraldehyde-3-phosphate**  **dehydrogenase (*GAPDH*)** | 4352934 | TaqMan |
| **Beta-2 microglobulin (*B2M*)** | HS_00187842_m1 | TaqMan |
| **NOTCH2 expression assays:** | | |
| **FL-NOTCH2** | *Ex33F:* AAAAATGGGGCCAACCGAGAC  *Ex34R:* TTCATCCAGAAGGCGCACAA | SYBR Green,  201bp |
| **TR-NOTCH2** | *Ex15F:* TGTGACAATCTGGTGAATGGA  *Ex16aR:* ACAGACCTGGGCACTGAGAC | SYBR Green, 250 bp |
| **Cloning primers:** | | |
| **FL-NOTCH2** | *FL_NOTCH2_ 5' Sgf1_F:* AATCGCGATCGCCATGCCCGCCCTGCGC  *FL_NOTCH2_3' PmeI_R:* GATTGTTTAAACCGCATAAACCTGCATGTTGTTG | 7415 bp |
| **TR-NOTCH2** | *TR_NOTCH2_ex1aF (external for cloning):*  TCAACAAAATGCCAAGTGGA  *TR_NOTCH2_5' Sgf1_F (internal for cloning):*  AATCGCGATCGCCATGCCCGCCCTGCGC  *TR_NOTCH2_3' PmeI_R:*  GATTGTTTAAACCGCATAAACCTGCATGTTGTTG | 2882 bp |
|  |  | 2592 bp |
| **BC1_CNV** | *BC1_CNV_F:* TGATGCAATTCTCAAGAACAGG  *BC1_CNV_R:* AAACTACCTGTTTCCCACTCCA | 101 bp |
| **BC2_CNV** | *BC2_CNV_F:* CTTATTCAAATCACGCCTTGC  *BC2_CNV_R:* CACCTCTCCTTTTCCCTTTTCT | 136 bp |

**Figure S1.** Expression of two splicing forms of *NOTCH2* in blood, tumor and normal breast tissue samples. Expression is shown as fold difference between *FL-NOTCH2* compared to *TR-NOTCH2*; p-values are for Student’s T-test between different sample sets.
